# Relation of CAD/CAM zirconia dental implant abutments with periodontal health and final aesthetic aspects; A systematic review

**DOI:** 10.4317/jced.59878

**Published:** 2023-01-01

**Authors:** Amin Davoudi, Kimia Salimian, Mahtab Tabesh, Bijan-Movahedian Attar, Mohammad Golrokhian, Meysam Bigdelou

**Affiliations:** 1 DDS, MSc. Assistant Professor, Department of Prosthodontics, Dental School, Shahrekord University of Medical Sciences, Shahrekord, Iran; 2 DDS. Dentist, private practice, Isfahan, Iran; 3 DDS. Research Assistant, Dental Research Center, Dental Research Institute, School of Dentistry, Isfahan University of Medical Sciences, Isfahan, Iran; 4 DDS, MSc. Professor, Dental Research Center, Dental Implants Research Center, Department of Oral and Maxillofacial Surgery, School of Dentistry, Isfahan University of Medical Sciences, Isfahan, Iran; 5 DDS, MSc. Post-graduate student, Department of Oral and Maxillofacial Surgery, School of Dentistry, Isfahan University of Medical Sciences, Isfahan, Iran; 6 DDS, MSc. Assistant Professor, Department of Oral and Maxillofacial Surgery, Dental School, Zanjan University of Medical Sciences, Zanjan, Iran

## Abstract

**Background:**

Using dental implants to replacing missing teeth and satisfy both functional and aesthetic needs is one of the mainstream dental treatments. New approaches including computer-aided design and computer-assisted manufacture (CAD/CAM) have been introduced to improve these elements. This systematic review aimed to compare CAD/CAM zirconia (Zr) implant abutments with other available abutments in terms of peri-implant health and aesthetics.

**Material and Methods:**

Five electronic databases (PubMed, Web of Science, Scopus, ProQuest, and Embase) were scoured for clinical studies evaluating Zr abutments reporting on the outcomes of interest including interproximal papilla stability (PS), papilla recession (REC), pink and white esthetic score (PES, WES), marginal bone level (MBL), color, and soft tissue contour. A hand searches in English language journals until September 2020 complemented the search. Two tools of Joanna Briggs Institute and Jaded Score calculation were used for the risk of bias assessment. No quantitative synthesis of the data was done due to high heterogeneity.

**Results:**

A total of six studies from the 412 ones obtained from the search were included. The study designs were either prospective cohort (n=3) or randomized clinical trial (n=3). Papilla fill, WES, PES, and the distance from the bone crest of adjacent teeth to the contact point (CPB) and inter-tooth–implant distance (ITD) was not significantly different between Zr CAD/CAM and Zr stock abutments. However, soft tissue stability and REC index were better in Zr CAD/CAM abutments.

**Conclusions:**

Higher soft tissue stability can be achieved for Zr compared to titanium abutments with either stock or CAD/CAM abutments.

** Key words:**Dental implants, Dental abutment, Computer-Assisted Design, Computer-Aided Manufacturing, Zirconia abutment, Soft tissue stability.

## Introduction

Replacing a missing tooth via a dental implant has become more common over the last decades (1). Nowadays, the factors that influence implant success extend beyond masticatory functional attributes (2), and are primarily based on aesthetic considerations. Dental aesthetics is one of the major components of facial attractiveness which has been associated with self-esteem, social success and even professional opportunities (3). Since the demand and expectation of patients for an aesthetic treatment have increased recently (4), satisfying the patients has become more challenging for clinicians. The clinicians should be able to rehabilitate gingival contour, especially papilla anatomy, to meet this desire (5), which is of great importance especially in the aesthetic zone.(6) New procedures and materials have been developed to improve the aesthetic aspects of implants (7). There are two main factors contributing to achieving optimal aesthetics: first, the clinician should use materials exhibiting the same color as natural teeth to avoid the grayish appearance of the overlying mucosa (8,9); second, the implant abutments should be appropriately contoured so that the surrounding soft tissue is properly supported (8,9). Therefore, the material that the implant abutment is manufactured from can have a noticeable impact on the aesthetics.

Various materials have been used to fabricate prosthetic implant abutments, such as metals, ceramics, and composites (10,11). According to modern prosthetic implant dentistry, customized abutments have become the centre of attention. They have two significant advantages: a) supporting surrounding soft tissue; b) a desirable cement margin location, so that the cement remnants can be removed sufficiently (1).

By computer-aided design and computer-assisted manufacture (CAD/CAM) technology, clinicians can individualize the shape and tilt of abutments required by its position. All information needed to make the final abutments is then transferred to a milling device (13). Using this technology makes it possible to compensate for the poor implant angulation, and finishing the abutment is under control (11,14). Progress in milling technology, especially CAD/CAM, can be used for preparing titanium (Ti) and zirconia (Zr). Ti is one of the preferred materials for implant abutments because of its satisfying mechanical properties like high strength, high resistance to distortion, and possibility of producing the abutment as one piece (15,16). Its major limitation was its dark color that can be seen through the soft tissue around the implant, producing a gray appearance (17). Whereas Zr abutment shows more acceptable aesthetic outcomes, especially for the zone with a thin gingiva biotype (18). Also, its low adhesion for bacteria and biocompatibility, besides its whitish color, made it aesthetically efficient (19,20).

This study systematically reviews the effect of CAD/CAM customized Zr dental implant abutments on peri-implant health and soft tissue aesthetics. The null hypothesis is defined as no significant differences between CAD/CAM customized Zr dental implant abutments and other available implant abutments in enhancing soft tissue health and aesthetic aspects.

## Material and Methods

To improve the structural reporting of articles, review arrangements were made by Guidelines for Preferred Systematic Reviews and Meta-Analysis Reports (PRISMA) (21).

The main aim of this review was to compare peri-implant health and aesthetics of CAD/CAM Zr implant abutments with other available abutments. The PICO was adjusted for eligible studies based on the following criteria: dental implants (P, population) with Zr CAD/CAM abutments (I, intervention) compared to other types of abutments (C, comparison) which affects the health and aesthetics of soft tissue (O, outcome). An advanced search was carried out by a Boolean search strategy using AND between the components of PICO and OR within each component (Table 1).

**Table 1:**
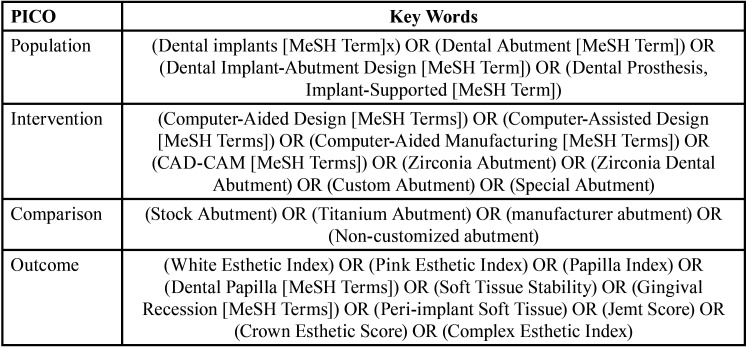
Applied keywords for the search including both MeSH and non-MeSH terms.

We found published articles by searching through the electronic database of PubMed, Web of Science, Scopus, ProQuest, and Embase until September 2020. We used Endnote software version 7 (Thomson Reuters, NY, USA) for purposes of cross-matching, and also to avoid missing data.

Two independent reviewers (A.D. and K.S.) screened the articles for eligibility. All reports received in English were examined to select the studies, and their titles and abstracts were checked for relevance based on the defined inclusion and exclusion criteria (Table 2). Also, other review articles and the reference lists of from various similar studies were used to find relevant articles. In cases of any disagreement, a discussion was undertaken between the reviewers to reach a mutual agreement. Reviewers’ consensus was tested with the Cohen κ test using MedCalc software (MedCalc Software, Ostend, Belgium) (kappa score = 0.90).

**Table 2:**
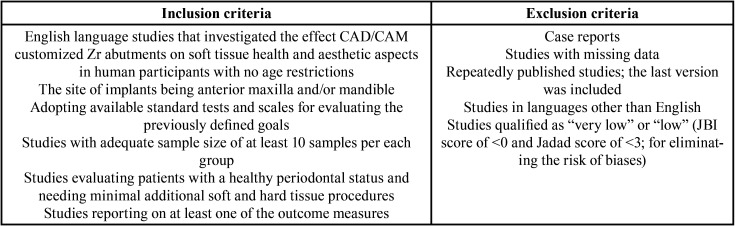
Inclusion and exclusion criteria.

The cohort studies were subjected to JBI (Joanna Briggs Institute) and the clinical trials were assessed based on Jadad Scores Calculation for the critical appraisal (Supplement 1,2) (https://www.medoral.es/medoralfree01/aop/jced_59878_s01.pdf)., (https://www.medoral.es/medoralfree01/aop/jced_59878_s02.pdf). (22,23). The full texts of selected studies were obtained according to the inclusion and exclusion criteria (Table 2).

Data were extracted from the selected studies using methods explicitly developed for data extraction. General information such as sample size and significant results were also collected. Each dispute was resolved through discussion, which resulted in consensus. No meta-analysis was prepared because the collected data were vastly heterogeneous (like different study designs with various sample preparation, different tests, and follow-up periods).

## Results

**Figure 1:**
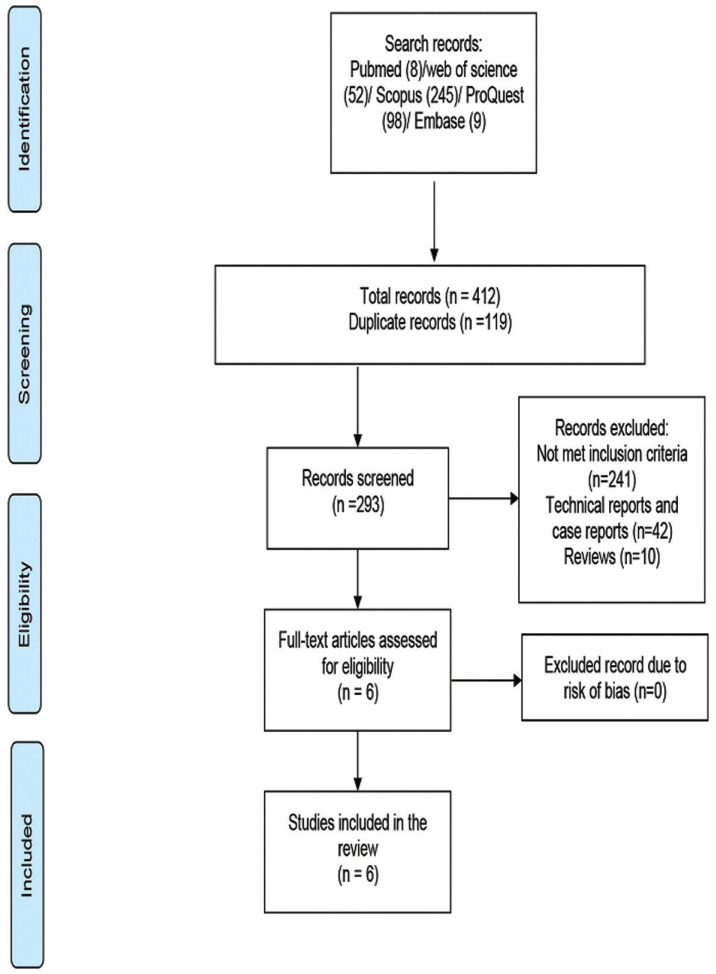
Flowchart of searching studies and database search.

A total of 412 articles were found after the initial search (8 on Pubmed, 245 on Scopus, 52 on Web of Science, and 98 on ProQuest, and nine on Embase). Two hundred ninety-three articles remained after the duplicate studies were removed, of which six studies (18,24-29) were eligible to be reviewed (Fig. 1). The full texts of these articles were retrieved and the relevant data were extracted based on the title/abstract. Three included studies were prospective cohort (25-27), and three were randomized clinical trials (Supplement 3) (https://www.medoral.es/medoralfree01/aop/jced_59878_s03.pdf). (24,28,29). Relying on the JBI, the three cohort studies had a score of 11(25-27), and according to Jadad scale the three clinical trials had a score of 6, all of which were considered of high quality (Supplement 1,2) (https://www.medoral.es/medoralfree01/aop/jced_59878_s01.pdf)., (https://www.medoral.es/medoralfree01/aop/jced_59878_s02.pdf). (24,28,29).

Combining the sample size from each study, a total of 304 implants were studied.

The implants have been placed in the anterior maxilla in three studies (24,25,29), while in other studies, they have been placed either in the anterior maxilla or mandible (Supplement 3) (https://www.medoral.es/medoralfree01/aop/jced_59878_s03.pdf). (26,28).

When analyzing the types of crowns, two studies used porcelain fused to metal (PFM) crowns (26,27), three studies used Zr ceramic crowns (24,26,27), one study applied lithium disilicate (IPS e.max) (25), one study used veneering ceramic (IPS E.max) (29). One study involved resin nano ceramic (Lava) (Supplement 3) (https://www.medoral.es/medoralfree01/aop/jced_59878_s03.pdf). (28).

Among these studies, the longest follow-up period (10 years) was conducted by Amorfini *et al*. (24) Two of the included studies have a 2-year follow-up (26,27). The other studies have one-year follow-up (Supplement 3) (https://www.medoral.es/medoralfree01/aop/jced_59878_s03.pdf). (25,28,29).

Supplement 4 (https://www.medoral.es/medoralfree01/aop/jced_59878_s04.pdf). summarises the measured outcomes in the included papers. One study assessed the interproximal papilla stability (PS) and papilla recession (REC) (27), three studies were on pink esthetic score (PES), white esthetic score (WES) and marginal bone level (MBL) (24,28,29), two evaluated the gingival margin (26,28), while only one study reported on soft tissue color and contour, and texture of soft tissue surrounding the implant (28).

The effect estimates have been detailed in Supplement 4 (https://www.medoral.es/medoralfree01/aop/jced_59878_s04.pdf). Since the heterogeneity of the data were high, only a qualitative synthesis of the data was performed and meta-analysis was not done.

## Discussion

The present review aimed to survey the influence of CAD/CAM Zr abutments on peri-implant health and soft tissue aesthetics criteria. Based on the reviewed studies, the defined null hypothesis was partially rejected.

The soft tissue improvement around implants is influenced by many factors including initial soft tissue thickness / soft tissue grafting procedures, bucco-palatal angulation of the implant and the prosthesis, vertical implant position, and the proximal bone level of the neighboring teeth (28). Once, the standard stock abutments were the only option available for the clinicians. However, there was growing evidence suggesting potential problems with them (30-32). These abutments might result in a round shape of the mucosa, with an unnatural appearance along with a concern of improper cement remnants removal (33-35).

Zr is considered a more acceptable material than Ti as an abutment for the soft tissue (20). Zr abutments make a better color appearance. Moreover, the polished Zr surfaces may lead to better adhesion for epithelial cells, decreasing the periodontal probing depths (36). Noticeably the level of bacterial load has been reported to be much higher in Ti abutments than Zr (37). According to Payer *et al*., (38) PES in Zr abutments was significantly higher than Ti abutments. Also, Linkevicius *et al*. (18) concluded that Zr abutments were more appropriate for abutment fabrication.

The development of new technologies such as CAD/CAM has attributed to improved aesthetic results. CAD/CAM allows the clinician to consider all aspects of geometry, including the outline of roots of adjacent natural teeth and the margin of gingiva (11,14). Recently, CAD/CAM technology with high precision and accuracy has been used to mill and customise Zr abutments. Based on Lops *et al*.’s study (26) in 2015, soft tissue stability was better in Zr and Ti CAD/CAM abutments than Zr and Ti stock abutments. For assessing soft tissue integrity, they evaluated the buccal gingival margin stability and the mean REC (26). While the gingival height around these implants varied from 0 to 1 mm over a 2-year observation period, a mean REC of 0.3 mm was determined for both Zr and Ti stock abutments with no significant differences (26). Inversely, slight soft tissue stability was observed only for Ti CAD/CAM abutments compared with stock Ti abutments (26). Even though the best outcomes for soft-tissue stability were shown by CAD/CAM Ti abutments, CAD/CAM Zr abutments might be desirable for thin buccal soft tissue in anterior areas to avoid the risk of soft tissue discoloration (26). Schepke *et al*. (28) considered stock abutments compared to CAD/CAM customized Zr implant abutments in another included study. No significant differences was seen in the position of the labial margin in these two types (28). No differences were reported in papilla fill and other clinical and radiographic parameters (28). Also, in another study by Lops *et al*. (27) in 2017, the mean REC index for restorations that were supported by stock abutments was higher than those with CAD/CAM abutments for both Ti and Zr. Small papilla regrowth was estimated for Zr and Ti CAD/CAM abutments.(27) Borges *et al*. (25) designed a study on customized CAD/CAM abutment using Zr and gold-titanium customized abutments versus custom metal abutments using a casting component. Despite the lack of difference among the known factors that can affect the papilla presence (such as the distance from the contact point to dental crest bone of adjacent tooth (CPB) and inter-tooth–implant distance (ITD)) between the two groups, an enhanced papilla presence was observed in the CAD/CAM group. It was concluded that the preferred outcomes for the CAD/CAM group was related to the proper crown contours and peri-implant soft tissue support (25). Overall, there seems to be a potential for CAD/CAM abutments to improve the soft tissue stability around the implants. Nevertheless, more studies are needed to reveal all the underlying factors that have led to inconclusive results so far.

Relying on biological aspects of using CAD/CAM Zr abutments, Amorfini *et al*. (24) conducted a 10-year follow-up study (24). In general, the results demonstrated no significant differences between biologic parameters such as periodontal probing depth (PPD), modified plaque index (mPI), and bleeding on probing (BOP) in Zr abutment with ceramic coated Zr crown (group ZrC), and CAD/CAM Zr abutment with ceramic fused to the abutment (group FCA) (24). After ten years, the mean MBL was significantly lower in the ZrC group (0.82 mm) than in the FCA group (0.95 mm) (24). The midfacial height of implant crown and height of the contralateral tooth crown values were similar in both groups (24). In most cases, the peri-implant papilla grew significantly in both groups in the first two years and was stabilized from the third year onward (24).

The type of chosen abutment can directly affect both PES and WES. PES deals with peri-implant soft tissue stability, and WES refers to the outcome of the crown itself (39,40). While WES improving involves developing new ceramics for abutments, the PES score mainly depends on the factors such as surgical process, implant characteristics, and loading protocols (41,42). Wittneben *et al*. (29) represented that the restoration method did not influence WES and PES. No statistically significant changes, including discoloration, were found in peri-implant mucosa over the observation period (29). Also, minimal crestal bone loss was observed in both prefabricated and CAD/CAM Zr groups (29). The main limitation of the current study was the inadequate number of studies along with high heterogeneity among them, which made it unjustifiable to conduct a meta-analysis.

## Conclusions

According to the included studies following conclusions can be drawn: CAD/CAM Zr abutments can enhance soft tissue stability and decrease the REC index. However, no difference is expected between CAD/CAM and stock abutments in WES, PES, CPB, ITD, and papilla fill. Secondary factors such as ease of manufacture, access to software, preference, or cost can also influence the selection between stock and a CAD/CAM customized Zr implant abutment. Since the studies present in this area are not consistent, more investigation must be done.
